# A new association of multiple congenital anomalies/mental retardation syndrome with bradycardia-tachycardia syndrome: a case report

**DOI:** 10.1186/1752-1947-3-9309

**Published:** 2009-12-01

**Authors:** Chinnamuthu Murugesan, Pradeep Kumar, Kanchi Muralidhar

**Affiliations:** 1 Department of Anesthesia, Narayana Hrudayalaya Institute of Medical Sciences, Bangalore, India

## Abstract

**Introduction:**

Congenital bradycardia-tachycardia syndrome is a rare disorder. Its association with multiple congenital anomalies/mental retardation (MCA/MR) syndrome is exceptional.

**Case presentation:**

We report a case of a new association of MCA/MR with bradycardia-tachycardia syndrome in an 18-year-old Indian man. This syndrome is characterized by mental retardation with delayed development of milestones, progressive scoliosis, cryptorchidism, asymmetrical limbs involving both the upper and lower limbs, sleep apnea syndrome, bradycardia-tachycardia syndrome and Dandy-Walker syndrome. Our patient was admitted for septoplasty with adenoidectomy. Patients with MCA/MR with bradycardia-tachycardia syndrome pose a unique challenge to the anesthesiologist. Establishing a good rapport with these patients is imperative. In addition to that, the anesthesiologist should anticipate the difficulty in intubation and rhythm abnormalities during the peri-operative period. Bradycardia or sinus arrest is a well-known complication during the induction and maintenance of anesthesia. Lignocaine should be used with caution in patients with bradycardia-tachycardia syndrome. Monitoring of ventilation parameters (end-tidal CO_2_, SPO_2_, airway pressure) is essential as these patients are prone to develop pulmonary artery hypertension secondary to sleep apnea syndrome.

**Conclusion:**

Based on our clinical experience in detailed pre-operative evaluation and planning, we would emphasize peri-operative anticipation and monitoring for dysrhythmias in patients with MCA/MR and bradycardia-tachycardia syndrome undergoing any surgical procedure.

## Introduction

Congenital bradycardia-tachycardia syndrome is a rare disorder [1]. Its association with multiple congenital anomalies/mental retardation (MCA/MR) syndrome is exceptional. We report a new association of MCA/MR with bradycardia-tachycardia syndrome. Anesthetic management in these patients is challenging and it requires careful pre-operative evaluation and planning, and adequate peri-operative monitoring is essential.

## Case presentation

**Figure 1:**
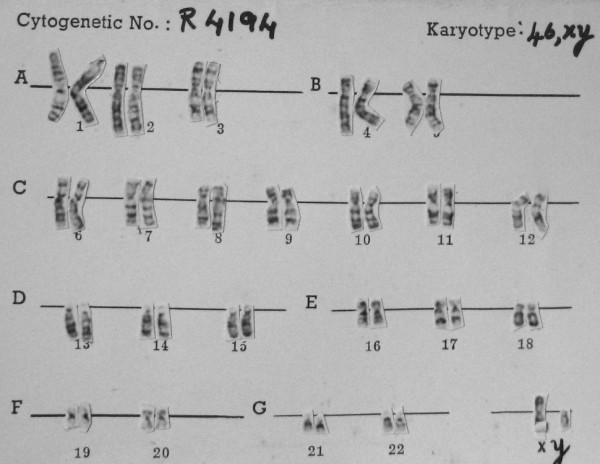
**Chromosomal study returning normal results**.

An 18-year-old Indian man was admitted for septoplasty with adenoidectomy. He was diagnosed as having MCA/MR syndrome, characterized by mental retardation with delayed development of milestones, progressive scoliosis, cryptorchidism, asymmetrical limbs that involve both the upper and lower limbs, supernumerary nipples (five), progressive myopia, obesity, sleep apnea syndrome, bradycardia-tachycardia syndrome and Dandy-Walker syndrome. His chromosomal study performed at the age of 5 was unremarkable (Figure 1).

**Figure 2:**
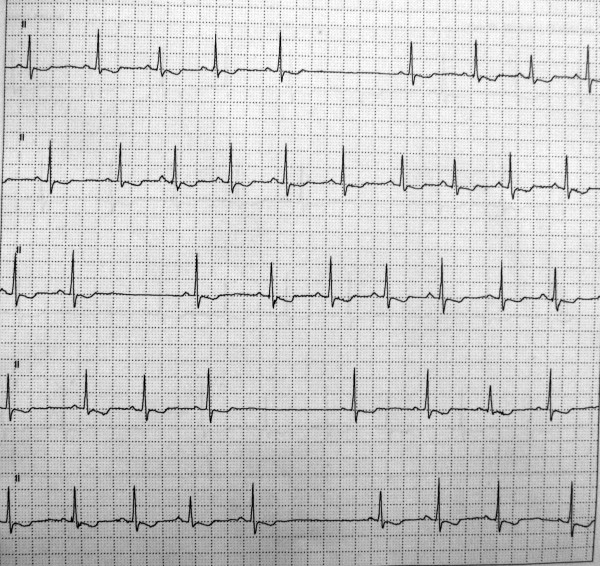
**Bradycardia-tachycardia syndrome with sinus pauses on 24-hour Holter monitoring**.

He was diagnosed as having bradycardia-tachycardia syndrome at the age of 6 months. A pediatric cardiologist advised the fitting of a permanent pacemaker as he had recurrent episodes of syncopal attacks, but his parents refused permission. According to them, syncopal attacks were transient in nature, lasting for a few seconds and clearing without any active medical intervention. A recent 24-hour Holter electrocardiogram (ECG) monitoring revealed bradycardia-tachycardia syndrome with sinus pauses (Figure 2). Of late, he had developed recurrent upper airway obstruction with excessive snoring during sleep. He was diagnosed as having a deviated nasal septum and enlarged adenoids for which he was advised to undergo septoplasty with adenoidectomy. In view of the upper airway obstruction caused by deviation of the nasal septum and hypertrophied adenoids, the treating physician suggested that this operation would definitely benefit this patient.

**Figure 3:**
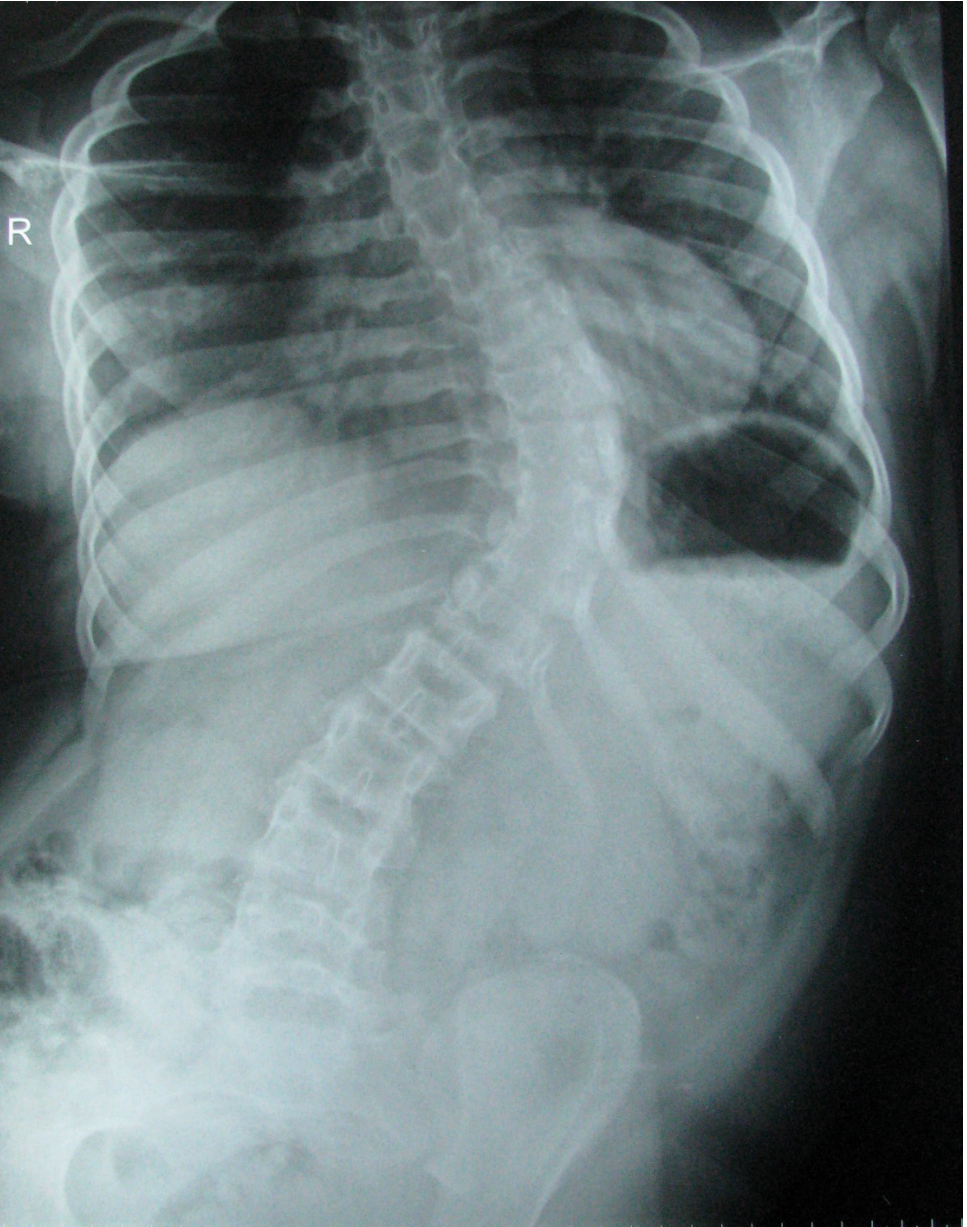
**Scoliosis of thoracic and lumbar spine**.

Radiography of his chest and abdomen showed scoliosis involving the thoracic and lumbar spine (Figure 3). There was restricted excursion of movements of the right hemithorax during inspiration. In addition, he was found to have been suffering from sleep apnea syndrome (SAS) for the past 6 months. However, polysomnography could not be performed as he was highly uncooperative. Arterial blood gas revealed PaO_2 _of 93 mmHg and PaCO_2 _of 34 mmHg with room air. Echocardiography showed normal biventricular function with tricuspid regurgitation and with a systolic gradient of 30 mmHg across the tricuspid valve. His pre-operative blood investigations including hemoglobin, liver function tests, thyroid function test and serum creatinine were within normal limits.

The patient was categorized as American Society of Anesthesiology (ASA) class 3, and general anesthesia was administered for the proposed surgery. Non-invasive transcutaneous pacemaker (NTP) paddles (Marquette defibrillation/pacing/monitoring pads, GE Medical Systems, Milwaukee, WI, USA) were attached to his chest wall. Anesthesia was induced with propofol and fentanyl; tracheal intubation was achieved with rocuronium. A 7.0 mm cuffed endotracheal tube was inserted into his trachea without any difficulty. Anesthesia was maintained with an inhalational mixture of O_2 _+ N_2_O (50:50) and isoflurane (1%). Standard intra-operative monitoring was adopted which included 5-lead ECG, non-invasive blood pressure, ETCO_2_, SPO_2 _and airway pressure. A 5.0 F sheath was inserted into the right internal jugular vein following induction of the anesthesia enabling insertion of the transvenous-pacing catheter in the event of bradycardia. Hemodynamic parameters were maintained within normal limits during the intra-operative period. Recovery from anesthesia was uneventful and his trachea was extubated at the end of the procedure. Postoperatively, the patient was intensively monitored for rhythm abnormalities. In the intensive care unit, he developed a fall in heart rate to 78/minute with sinus pauses, which was effectively treated with intravenous atropine. However, he did not require the temporary pacing during the peri-operative period. He was discharged from hospital 2 days later. During the follow-up period 1 month after the operation, the patient showed symptomatic improvement in upper airway obstruction.

## Discussion

Bradycardia-tachycardia syndrome usually reflects the presence of sinoatrial disease, where episodes of supraventricular tachycardia complicate sinus bradycardia with or without periods of sinus arrest or sinoatrial block. Dizziness, syncope or convulsions may result from cerebral ischemia secondary to bradycardia, and tachycardia may cause palpitation, dyspnea and chest pain. The etiology of this syndrome is not known, but associations with coronary artery disease, thyrotoxicosis, cardiomyopathy, amyloidosis, diabetes and cardiac surgery have been reported [2]. However, to the best of our knowledge, its association with MCA/MR syndrome has not been described in the literature.

We report the case of a patient with MCA/MR syndrome with bradycardia-tachycardia syndrome (probably congenital) scheduled for septoplasty with adenoidectomy. Patients with MCA/MR with bradycardia-tachycardia syndrome pose a unique challenge to the anesthesiologist. Establishing a good rapport with these patients is imperative. Anesthetic considerations include a) anticipation of difficulties in intubation due to restricted neck movements, short neck, and tracheal deviation; b) rhythm abnormalities during the peri-operative period; and c) adequate attention towards skeletal abnormalities, for example, scoliosis, as evident in this patient.

Bradycardia or sinus arrest is a well-known complication during the induction and maintenance of anesthesia [3]. This issue can be overcome by placing a temporary transvenous pacemaker [4] or with the use of NTP [5]. Hemodynamic instability caused by sinus tachycardia or supraventricular tachycardia during the intra-operative period can be treated either with cardioversion or pharmacological measures [6]. Lignocaine should be used with caution in patients with bradycardia-tachycardia syndrome. It is presumed that lignocaine directly depresses the sinus node automaticity in these patients [7].

Our patient had been diagnosed previously as having SAS. Patients with SAS are sensitive to all central depressant drugs, with upper airway obstruction or respiratory arrest occurring even with a minimal dose of sedatives, hypnotics or narcotics [8]. Anesthetic drugs should be administered by titration to the clinical effects, preferably using short-acting drugs. In one study, it was found that the prevalence of SAS is ten-fold higher in patients with bradycardia-tachycardia syndrome than in the general population. This observation reveals that there may be a relationship between these two syndromes [9].

Monitoring of ventilation parameters (ETCO_2_, SPO_2_, airway pressure) is essential as these patients are prone to develop pulmonary artery hypertension secondary to SAS [7] or scoliosis. In our patient, echocardiography revealed tricuspid regurgitation with a systolic pressure gradient of 30 mmHg, which indicates mild pulmonary artery hypertension.

## Conclusion

Based on our clinical experience, we emphasize the importance of detailed pre-operative evaluation and planning, and peri-operative anticipation and monitoring for dysrhythmias in patients with MCA/MR and bradycardia-tachycardia syndrome undergoing any surgical procedure.

## Abbreviations

MCA/MR: multiple congenital anomalies/mental retardation; ECG: electrocardiogram; SAS: sleep apnea syndrome; ASA: American Society of Anesthesiology; NTP: non-invasive transcutaneous pacemaker.

## Consent

Written informed consent was obtained from the patient's parents for publication of this case report and any accompanying images. A copy of the written consent is available for review by the Editor-in-Chief of this journal.

## Competing interests

The authors declare that they have no competing interests.

## Authors' contributions

CM provided patient care, acquisition of data and literature review. KM was involved in drafting the manuscript. PK was involved in patient care and interpretation of data.
